# Identification of Potential Therapeutic Drugs for Huntington's Disease using *Caenorhabditis elegans*

**DOI:** 10.1371/journal.pone.0000504

**Published:** 2007-06-06

**Authors:** Cindy Voisine, Hemant Varma, Nicola Walker, Emily A. Bates, Brent R. Stockwell, Anne C. Hart

**Affiliations:** 1 Massachusetts General Hospital Cancer Center, Charlestown, Massachusetts, United States of America; 2 Department of Biological Sciences, Columbia University, Fairchild Center, New York, New York, United States of America; 3 Department of Chemistry, Columbia University, Fairchild Center, New York, New York, United States of America; 4 Harvard Medical School Department of Pathology, Boston, Massachusetts, United States of America; Emory University, United States of America

## Abstract

**Background:**

The prolonged time course of Huntington's disease (HD) neurodegeneration increases both the time and cost of testing potential therapeutic compounds in mammalian models. An alternative is to initially assess the efficacy of compounds in invertebrate models, reducing time of testing from months to days.

**Methodology/Principal Findings:**

We screened candidate therapeutic compounds that were identified previously in cell culture/animal studies in a *C. elegans* HD model and found that two FDA approved drugs, lithium chloride and mithramycin, independently and in combination suppressed HD neurotoxicity. Aging is a critical contributor to late onset neurodegenerative diseases. Using a genetic strategy and a novel assay, we demonstrate that lithium chloride and mithramycin remain neuroprotective independent of activity of the forkhead transcription factor DAF-16, which mediates the effects of the insulin-like signaling pathway on aging.

**Conclusions/Significance:**

These results suggest that pathways involved in polyglutamine-induced degeneration are distinct from specific aging pathways. The assays presented here will be useful for rapid and inexpensive testing of other potential HD drugs and elucidating pathways of drug action. Additionally, the neuroprotection conferred by lithium chloride and mithramycin suggests that these drugs may be useful for polyglutamine disease therapy.

## Introduction

Drug discovery for late onset neurodegenerative diseases is a major challenge. In large part, the complexity of treating these disorders results from our insufficient understanding of the contributions of multiple pathways on disease pathophysiology. Furthermore, since the pathology of these disorders is often only discernable in aged populations, testing the therapeutic value of small molecules in vertebrate disease models requires time consuming and costly experimental designs. The development of rapid and inexpensive *in vivo* assays to evaluate the numerous candidate compounds identified in high-throughput screens is therefore of paramount importance.

Invertebrate model organisms such as *C. elegans* provide an attractive alternative for prioritizing lead compounds in the early stages of drug development for age-related diseases [1]–[3]. *C. elegans* has several characteristics that make it ideal for drug testing- including a short lifecycle, small size and the ease of culturing in liquid. Furthermore, decades of neurobiological and antiparasitic drug studies in *C. elegans* provide a strong foundation for use of this organism in therapeutic compound identification [1]–[3].

Huntington's Disease (HD) is caused by expansion of a polyglutamine (polyQ) tract in the huntingtin protein leading to neurodegeneration that is age and polyQ tract length dependent [4]. In this study, we use a *C. elegans* model of polyQ neurotoxicity in which the N-terminal 171 amino acid fragment of human huntingtin protein containing an expanded polyglutamine tract (150Qs) is expressed in neurons. Degeneration and cell death in this model is dependent on both age and polyglutamine tract length, recapitulating these aspects of the human disease [4]–[6].

We tested a collection of compounds that have been previously described to decrease degeneration in cell culture/animal models of polyQ toxicity for their ability to protect *C. elegans* neurons from the toxic effects of an expanded huntingtin polyglutamine fragment. We developed, optimized and validated new assays for use in rapid assessment of drug efficacy using *C. elegans* HD models. Of the compounds tested, we found that two FDA approved drugs, mithramycin (MTR) and lithium chloride (LiCl), reduced polyQ toxicity in the *C. elegans* model.

An important determinant of neurodegenerative diseases is the aging process. However, the mechanistic links between aging and the cellular pathways leading to neurodegeneration are not well understood. The forkhead transcription factor DAF-16, which mediates the effects of the insulin-like signaling pathway on aging, has been shown to play a role in polyQ aggregation. Mutations that reduce insulin signaling, derepress DAF-16 leading to an increase in lifespan and stress resistance [7], [8], whereas RNAi based knockdown of *daf-16* accelerates polyQ aggregation and toxicity [9] suggesting that DAF-16 transcriptional targets not only promote longevity but also prevent polyQ aggregation. Despite the pivotal role that growth and aging play in neurodegenerative disease, we found that LiCl and MTR protect *C. elegans* neurons in the absence of growth and through a *daf-16* independent pathway suggesting that these compounds may target pathways that are specific to neurodegeneration. Thus, the integration of pharmacological and genetic examination of drugs, in *C. elegans* HD models that we describe, should accelerate the identification of interventions for HD along with insight into mechanism of drug action.

## Results

### Compound concentration range for screening in *C. elegans*: Food Clearance Assay

**Figure 1:**
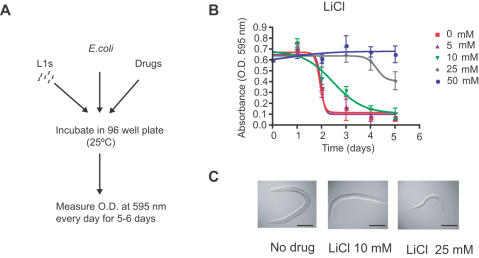
Concentration of compounds for testing was assessed using a food clearance assay. (A) Flow diagram of the food clearance assay. 20 newly hatched L1 animals were incubated at 25°C in *E. coli* at a final OD (A_595_) of 0.6 in 96 well microtiter plate wells containing varying drug concentrations. The OD of the microtiter plate was measured daily for 5 days. (B) The OD of *E. coli* is reported daily for each concentration of LiCl. The mean OD is calculated for each day from triplicate samples and plotted over time. Error bars represent SEM. Food clearance assays were also performed on trichostatin A and mithramycin, (Figure S1). (C) Animals treated with no drug and indicated LiCl concentrations. Animals treated with 50 mM or 100 mM LiCl are alive but concentrations above 100 mM LiCl cause death (data not shown). Scale bar is approximately 75 µm.

We tested a collection of compounds that have demonstrated therapeutic value in either cell culture and/or animal models of polyQ toxicity for screening in our *C. elegans* HD models (**Table 1**). These candidates represent compounds that may protect against polyQ toxicity by affecting a variety of cellular pathways. To efficiently evaluate the effects of these candidates on neurodegeneration and neuronal cell death, we first established a systematic method for selecting optimal drug concentrations to assess in our *C. elegans* HD models (see Methods). Compounds were tested in a dose dilution series in the food clearance assay (**Figure 1** and **Figure S1**) starting at the highest soluble concentration. Taking advantage of the short life cycle and the ability of *C. elegans* to grow in liquid culture of *E. coli*, we evaluated compounds by monitoring the rate at which the *E. coli* suspension (food source) was consumed. Each adult is capable of producing hundreds of progeny that rapidly consume the limited *E. coli* supply. As a result, the OD of wells without compound drastically decreases in 3 days. Any drug that decreases *C. elegans* growth, survival or fecundity would result in a dose dependent reduction of the rate at which food is cleared (consumed) in a well. For example, addition of 5 mM LiCl to the culture showed no effect on food clearance compared to control animals, whereas animals exposed to 10 mM or 25 mM LiCl had delayed food clearance (**Figure 1B**). Visual inspection confirmed that animals treated with 10 mM and 25 mM LiCl were smaller in size compared to untreated animals whereas animals treated with 5 mM LiCl were unaffected (**Figure 1C** and data not shown). Furthermore, animals exposed to 50 mM and 100 mM LiCl did not produce progeny over the time course of the experiment (data not shown), which correlated with the lack of clearance of the *E coli* food source. For all compounds, optimal concentrations for *C. elegans* were similarly assessed using the food clearance assay (**Table 1**). This simple assay for determining a compound's concentration range to test in *C. elegans* uses a small amount of compound and is amenable to high-throughput format, making it applicable to any *C. elegans* drug study.

**Table 1:** Compounds screened in *C. elegans* polyQ model for neuroprotection.

Drugs tested	Putative Mechanism	TC 50 Food clearance (mM)	Conc. Tested (mM)	EC 50 (mM)	Rescue in *C. elegans*	Activity reported in HD model(s)
LiCl	GSK-3β inhibitor	25	10–100	25	Yes	Cell culture [31] *D. melanogaster* [32]
Mithramycin	DNA binding	0.003	0.01–1	0.5	Yes	*M. musculus* [33]
Trichostatin A (TSA)	HDAC inhibitor	1	0.16–1	0.16	Yes	Cell Culture [34]
						C. elegans [14]
						S. cerevisiae [35]
SAHA*	HDAC inhibitor	2	2	n.a.	Yes	M. musculus [36]
						D. melanogaster [37]
Taxol	Cytoskeletal dynamics	none	0.4	n.a.	no effect	Cell culture [38]
Congo Red	Aggregation inhibitor	0.5	0.12–0.5	n.a.	no effect	*M. musculus* [39]
Cystamine	Transglutaminase inhibitor	300	150	n.a.	no effect	M. musculus [40]–[42]
						D. melanogaster [43]
						Cell culture [44]
Budesonide	Glucocorticoid receptor agonist	none	0.8	n.a.	no effect	Cell culture [45]
Paraquat	Free radical generator	3	3	n.a.	no effect	Negative control [46]

All compounds were tested in *pqe-1* enhanced background in the presence of food, *E. coli*.

n.a.  =  Not applicable as these compounds were not active.

* SAHA was only tested and found to be active at a single concentration (2 mM). Therefore, an EC50 was not determined.

### Screening neuroprotective compounds in *C. elegans* polyQ model

In order to test compounds in an efficient manner, we decided to reduce the amount of time required to test drug efficacy. Relatively long assay duration may also be unsuitable for testing compounds that are unstable under assay conditions. We utilized a genetic mutant background (*pqe-1* loss of function) that accelerates polyQ mediated neurodegeneration and cell death in *C. elegans* from 7 days to 2–3 days. We evaluated neuronal death by monitoring loss of expression of a GFP reporter construct in the bilateral ASH sensory neurons in *pqe-1;Htn-Q150* animals, a genetically enhanced model of polyQ toxicity [10] (**Figure S2**). In *pqe-1;Htn-Q150* animals, the vast majority (>90%) of ASH neurons undergo cell death in less than three days (**Figure S2**).

**Figure 2:**
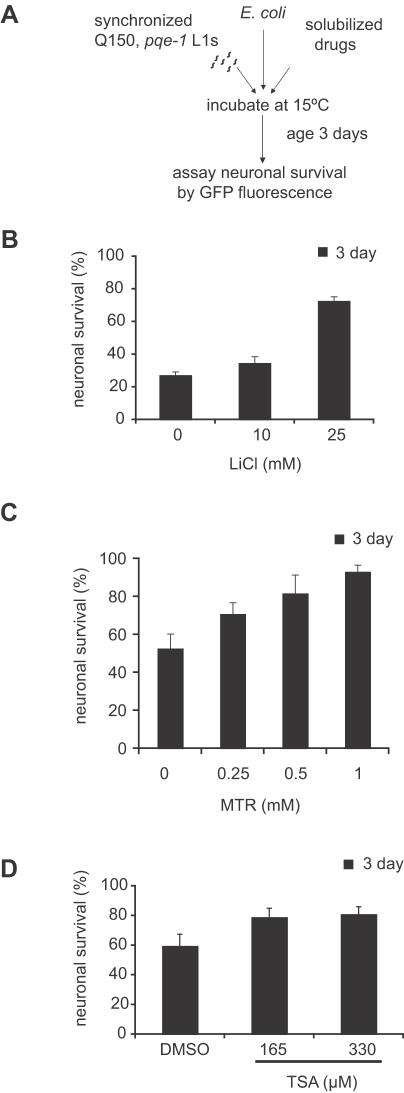
LiCl, TSA, and MTR decrease polyQ mediated ASH neuronal death. (A) Flow diagram of the sensitized assay. 20–30 synchronized *pqe-1;Htn-Q150* L1s per well were incubated at 15°C in S medium with *E. coli* at an OD (A_595_) of 0.6 and varying concentrations of compound to a total volume of 60 µl. Animals were grown in the presence of compound for 3 days. ASH neuron survival was evaluated by the presence or absence of GFP expression. (B–D) LiCl, TSA and MTR increased ASH neuron survival in the *pqe-1;Htn-Q150* animals in the presence of *E. coli*. ASH neuron survival was evaluated by the presence or absence of GFP expression on day 3. 100 neurons were scored for each trial; mean percentage of 3 different trials±SEM is shown. p<0.0001 for 25 mM LiCl. p = .037 for 0.5 mM MTR; p<0.0001 for 1 mM MTR. p = 0.0082 for 165 µM TSA; p = 0.005 for 330 µM TSA.

Synchronized *pqe-1;Htn-Q150* L1 larvae were treated with and without drug for three days, then GFP expression was monitored to assess ASH neuronal viability (**Figure 2A**). Of the candidates tested, we identified three protective compounds, mithramycin (MTR), trichostatin A (TSA) and lithium chloride (LiCl) (**Table 1**). All three compounds caused a dose-dependent neuroprotection in the *pqe-1* sensitized background (**Figure 2B**). MTR and TSA affect transcriptional activity [11], [12] and LiCl is a drug used clinically to treat individuals with bipolar disorders and is a potential inhibitor of GSKβ [13]. Previous studies in *C. elegans* demonstrated that TSA reduced polyQ mediated neurodegeneration [14], validating this approach. Htn-Q150 transgene expression was not altered by drug treatment based on RT-PCR (**Figure S3**). Based on these results, we conclude that a genetically sensitized background (*e.g. pqe-1*) can be used to dramatically reduce the amount of time required to accurately assess effects of drugs on neuronal cell death.

### Neuroprotection in an age-dependent *C. elegans* polyQ model

To determine if neuroprotection was dependent on *pqe-1*, we tested candidates in the Htn-Q150 only background that exhibits age-dependent neurodegeneration but no cell death. Late onset degeneration of ASH sensory neurons was monitored by defects in dye uptake, a previously described assay [6] (**Figure S2**).

One limitation due to the prolonged duration (7 days) of the aging assay is the production of progeny (200–300 per adult) by the aging animals. Since the progeny grow to adulthood in 3 days, they are difficult to distinguish from the aged parent by day 7. A novel genetic strategy was employed to prevent progeny development. We crossed Htn-Q150 into a genetic background (*pha-1*) in which progeny fail to feed and grow at the restrictive temperature of 25°C [15]. Introducing *pha-1(e2123)* to eliminate progeny development permits the use of liquid cultures and facilitates any *C. elegans* aging study (see Methods).

**Figure 3:**
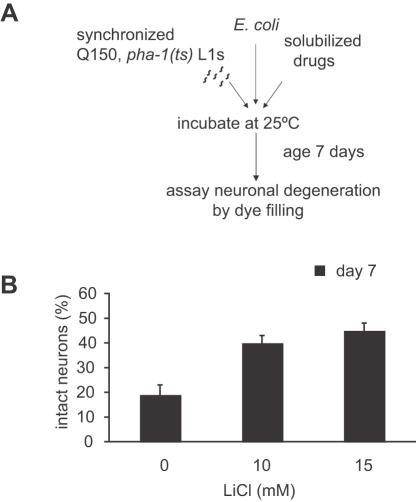
Compounds decrease polyQ toxicity in a *pqe-1* independent manner in aged animals. (A) Flow diagram of the aging assay. 30 synchronized *pha-1;Htn-Q150* L1s per well were incubated at 25°C in S medium with *E. coli* at an OD (A_595_) of 0.5 with varying concentrations of compound to a total volume of 1 ml. On day 7, animals were collected and dye filled using DiD. (B) LiCl increased ASH neuron survival in the *pha-1;Htn-Q150* aged animals. ASH neurodegeneration was evaluated by the presence or absence of dye-filled ASH neurons on day 7. 50 neurons were scored for each trial; mean percentage of 3 different trials±SD is shown. p = 0.0033 for 15 mM LiCl.

To assay compound efficacy, synchronized *pha-1;Htn-Q150* L1 larvae were treated with and without drug for seven days, then scored for degeneration using the dye-filling assay (**Figure 3A**). Introducing the *pha-1* mutation had no effect on the dye-filling of ASH neurons or the polyQ dependent neurodegeneration (data not shown and **Figure S2**). LiCl decreased polyQ neurodegeneration in a dose dependent manner (**Figure** **3B**) while equivalent concentrations of sodium chloride (NaCl) had no effect (data not shown), showing the specificity of LiCl's effect. Consistent with previous findings [14], the HDAC inhibitor trichostatin A (TSA) also suppressed neurodegeneration in the aging assay format (**Figure S4**). The confirmation of compound activity in the age dependent Htn-Q150 model validated the utilization of the *pqe-1* sensitized strain as a rapid means of identifying HD therapeutic compounds.

### Dissociating neuroprotection from growth, development and aging

**Figure 4:**
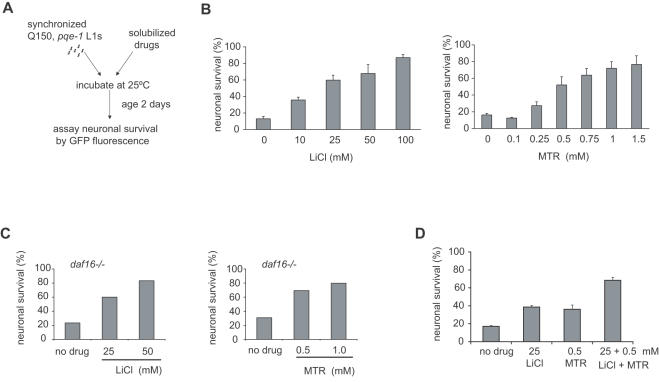
LiCl and MTR decrease polyQ toxicity measuring ASH neuronal death in the absence of growth. (A) Flow diagram of the starvation assay. 50 synchronized *pqe-1;Htn-Q150* L1s per well were incubated at 25°C in S medium with varying concentrations of compound to a total volume of 60 µl. On day 2, the survival of the ASH neuron was determined by visually evaluating the presence or absence of GFP expression. (B) LiCl and MTR increase neuronal survival in starved conditions. 100 neurons were scored for each trial; mean percentage of 3 different trials±SEM is shown. p = 0.0063 for 10 mM LiCl; p<0.0001 for 25 mM, 50 mM and 100 mM LiCl. p<0.0001 for 0.5 mM, 0.75 mM, 1.0 mM, and 1.5 mM MTR. (C) LiCl and MTR increase neuronal survival in a *daf-16* independent manner. At least 100 neurons were scored for each trial; percentage of neuronal survival in one representative experiment of 2 independent trials is shown; p<0.0001 for 25 mM and 50 mM LiCl. p<0.0001 for 0.5 mM and 1.0 mM MTR. (D) Combined, MTR (0.5 mM) and LiCl (25 mM) were more effective than either drug alone in protecting ASH neurons from polyQ neurotoxicity. 50 synchronized *pqe-1;Htn-Q150* L1s per well were incubated at 25°C in S medium with 0.5 mM MTR and 25 mM LiCl to a total volume of 60 µl. On day 2, the survival of the ASH neuron was evaluated by the presence or absence of GFP. 100 neurons were scored for each trial; mean percentage of 3 different trials±SEM is shown. The excess synergy was 0.14 based on a Bliss Independence analysis. p = 0.046 for 0.5 mM MTR; p = 0.0039 for 25 mM LiCl; p<0.0001 for LiCl+MTR.

Since the aging process is a critical but poorly understood contributor to late onset neurodegenerative diseases [9], [16], [17], compounds that affect aging may indirectly affect degeneration. To distinguish drug effects on neuronal cell death from those on growth, development and aging rates, we applied two strategies: (i) we modified our assay format to prevent growth of animals for the duration of the assay (ii) we genetically altered the well characterized insulin signaling pathway that affects aging. To modify our assay, we took advantage of the growth arrest induced by starvation of L1 stage *C. elegans* larvae. When embryos hatch in the absence of food, their development is arrested until food is available [18], [19]. Since starved L1 animals do not grow, the effects of compounds on neuronal cell death can be evaluated independent of their effects on growth and development. A further advantage is that drugs can be tested in the absence of bacteria (*E. coli* food source) that could degrade or metabolize compounds under analysis. Using a starvation assay format (**Figure 4A**), we found that ASH neurons continue to undergo neuronal cell death in *pqe-1;Htn-Q150* animals that are growth arrested by the absence of food (data not shown). This finding suggests that certain pathways that affect polyQ neurodegeneration are distinct from pathways that effect growth and development.

We tested LiCl and MTR in this starvation assay format. Both LiCl and MTR protected ASH neurons of starved animals in a dose-dependent manner (**Figure 4B**). Equivalent concentrations of NaCl as that of LiCl had no effect on neuronal cell death demonstrating specificity of the compound's effect (data not shown). Using this assay modification, higher concentrations of compounds that interfere with *C. elegans* development and growth were tested in the starvation assay (**Figure 1C** and **Figure 4B**). Therefore, testing drugs in the L1 arrest assay can distinguish contributions of specific drugs on organismal development and aging, providing a clearer demonstration of the neuroprotective properties of compounds.

The insulin signaling pathway, a critical regulator of longevity in *C. elegans*, is also a modifier of polyQ toxicity. RNAi mediated knockdown of *daf-16* decreases lifespan and leads to early onset of polyQ aggregation [9]. To determine if the neuroprotective effects of LiCl and MTR are dependent on components of the insulin signaling pathway, we crossed a *daf-16* null mutation into *pqe-1;Htn-Q150* animals and tested the effects of LiCl and MTR on neuronal cell death in the starvation assay. We found that both compounds remained neuroprotective in the *daf-16* mutant animals in a dose dependent manner (**Figure 4C**), suggesting that LiCl and MTR suppress polyQ mediated neuronal cell death independent of DAF-16 activity. The dissociation of the neuroprotective effects of compounds from growth and specific aging pathways allows a mechanistic classification of therapeutic compounds.

### Combinatorial effects of LiCl and MTR

A number of therapies for complex diseases use combinations of drugs that allow targeting of multiple mechanisms and allow therapeutic efficacy at lower compound concentrations, limiting toxicity of single agents. We also investigated the utility of our *C. elegans* assay in drug combinatorial studies by testing LiCl in combination with MTR on *C. elegans* polyQ toxicity in the starvation assay. Combining 25 mM LiCl and 0.5 mM mithramycin in the starvation assay format increased ASH neuron survival compared to the protection afforded by either compound alone (**Figure 4D**).

## Discussion

**Figure 5:**
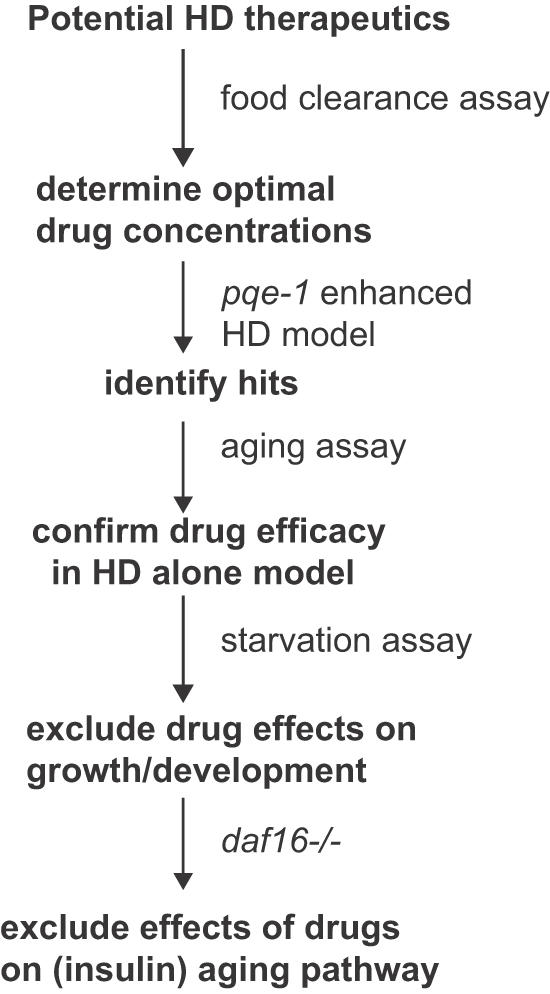
Schematic Diagram of Compound Testing Strategy. (1) A range of concentrations for each compound for testing in *C. elegans* was established using the food clearance assay. (2) The protective effects of compounds on polyglutamine neurodegeneration and cell death were assessed in *pqe-1;Htn-Q150* animals treated with compounds for 3 days. (3) To determine if neuroprotective effects of compounds were dependent on *pqe-1*, compounds were retested in animals expressing *Htn-Q150* for 7 days. (4) To distinguish drug effects on neuronal cell death versus effects on growth and/or development, synchronized *pqe-1;Htn-Q150* L1 animals were incubated in the presence of drugs without food for 2 days. (5) The neuroprotective effects of compounds on the aging process can be tested by introducing mutations (*daf-16*) in components of specific aging-related (insulin signaling) pathways.

In this study, we have devised strategies for efficiently assessing therapeutic efficacy of compounds in *C. elegans* HD models (**Figure 5**). Our methods exploit the strengths of *C. elegans* model for drug discovery, including convenient and precise visualization of live neurons, small-scale liquid cultures that significantly reduce the amount of drug required for testing as well as genetic approaches to implicate specific cellular pathways in a compound's mechanism of action. These assays could be utilized for prioritizing the large numbers of hits already identified by high-throughput screens for HD and other polyQ diseases [20]–[23].

The normal aging process is intricately linked to late onset neurodegenerative diseases. However, the influence of aging on cellular events that impact neurodegeneration is unclear. To dissect the interplay of pathways involved in neurodegeneration from those involved in aging, we combined genetic manipulations with a novel assay that utilizes growth-arrested animals. Our studies demonstrate that LiCl and MTR exert their neuroprotection in a *daf-16* independent manner. However, starvation of animals may activate additional stress responsive transcription factors such as heat shock factor (HSF1). HSF1 regulates the heat shock response and also influences longevity [17], [24], [25]. Furthermore, HSF1 RNAi enhances polyQ aggregation [26] raising the possibility that HSF1 or other stress activated pathways may function as molecular targets of these compounds. However, the fact that neurons continue to die under conditions of starvation suggests that the stress responses induced by starvation are insufficient to abrogate the neurodegenerative process. Thus, the combination of strategies outlined in the study allows the separation of drug effects on neurodegeneration from those on growth and stress related aging pathways.

Though we focus on neurodegeneration in HD models, similar strategies may apply to other age dependent phenotypic readouts. For example, deletion of the *C. elegans* dystrophin gene, the homolog of a gene involved in Duchenne's Muscular Dystrophy, in combination with a second mutation in the myogenic factor MyoD leads to progressive muscle degeneration [27], [28]. Using the strategies described in this study, the protective effect of compounds on muscle degeneration could be conducted rapidly. These *C. elegans* disease models should be valuable tools for rapid identification of potential therapeutic compounds for aging related human diseases and cellular pathway elucidation of any compounds that are active in these models.

## Materials and Methods

### Strains

*C. elegans* N2[wild-type] and GE24[*pha-1(e2123)*], *E. coli* food sources OP50 and HB101 (OP50 for compound efficacy and aging assays and HB101 for sensitized and food clearance assays) were obtained from the *Caenorhabditis elegans* Genetics Center. *Htn-Q150*(*rtIs11*) expressing strains with and without *pqe-1*(*rt13*) were previously described [6], [10]. *daf-16(mgDf47*) was crossed into *pqe-1(rt13*) mutant strain expressing *Htn-Q150*(*rtIs11*) marked with *dpy-11(e224)*. All mutations were homozygous in the final strain for analysis. All strains were cultivated on nematode growth media (NGM) plates at 15°C [29].

### Food Preparation

*E. coli* were grown overnight at 37°C in Luria broth (LB) media, pelleted by centrifugation, frozen at −70°C, and then resuspended at a final OD of 0.5 (595 nm) for the aging assay or an OD of 6.6 in nematode S-medium [29] (supplemented with 100× streptomycin/penicillin (Invitrogen, #15140-122) and nystatin (Sigma, #N1638)) for all other food based assays. Note the lower OD (∼0.6) in plates was due to a decreased path length of the 60 µl final suspension, in a well, compared to a 1 cm path length in a spectrophotometer.

### Compound Preparation

All compounds were purchased from Sigma (St. Louis, MO). Stock solution of 1 M lithium chloride (LiCl) in water, 10 mM mithramycin (MTR) in PBS and 5 mg/ml trichostatin A (TSA) in DMSO were stored at room temperature (LiCl), 10–14 days at 4°C (MTR) and at −20°C (TSA). Compounds were diluted into *E. coli* suspension or S-medium (starvation assay) to the desired concentrations. 1 ml of the final mixture was added per well of a 24-well polystyrene plate for the aging assay. For the remaining assays, 50 µl of the final mixture was added per well in a 96-well polystyrene plate.

### *C. elegans* Preparation

We used newly hatched *C. elegans*, collected as L1 larvae at 15°C. Specifically, adults were lysed, fertilized eggs were collected by hypochlorite treatment of gravid adults and suspended in S-medium for 24–30 hours at 15°C and L1 larvae were allowed to hatch overnight in the absence of food [29]. *pha-1(e2123)* is a temperature sensitive loss of function allele that affects normal pharyngeal development [15]. A normal pharynx forms at the permissive temperature of 15°C in newly hatched L1 animals while the pharynx fails to develop in *pha-1(e2123)* animals hatched at 25°C. *C. elegans* L1 animals do not initiate growth in the absence of food, resulting in a synchronous population of arrested L1 animals. Introduction of a food source (*E. coli*) results in growth; animals become adult hermaphrodites within 48 hours at 25°C. This L1 growth arrest and recovery provides an efficient means of collecting large numbers of synchronized animals for assays.

### Food Clearance Assay

The effect of compounds on *C. elegans* physiology is monitored by the rate at which the *E. coli* food suspension was consumed, as a read out for *C. elegans* growth, survival or fecundity. Approximately 20–30 L1 synchronized animals per 10 µl of S-media were added to an *E. coli* suspension. Microtiter plates containing animals and drugs were incubated at 25°C. The absorbance (OD 595nm) was measured daily using a Vmax Kinetic microplate reader (Molecular Devices). We used N2 animals when determining compound concentration. Introduction of *pha-1(e2123)*, *pqe-1(rt13), daf-16(mgDf47)* or *Htn-Q150(rtIs11)* had no effect on overall growth or health of the animal compared to N2 in the presence of drug at assay temperatures.

### Scoring Degeneration and Cell Death

At time points indicated, animals were collected by centrifugation, washed in S-media and immobilized with 5 mM sodium azide on a 2% agarose pad. GFP fluorescence was scored using an Axioplan2 fluorescence microscope (n = 100 ASH neurons). For the aging assay, degeneration was assessed by dye-filling [6]. Animals were incubated for 2 hours in 1,1′-dioctadecyl-3,3,3′,3′-tetramethylindodicarbocyanine perchlorate (DiD) (Molecular Probes) and immobilized as above for scoring ASH dye filling (n = 50 ASH neurons). To determine statistical significance, we compared the results from parallel experiments using 2×2 contingency tables comparing the number of GFP+ and GFP- neurons in control vs. specific concentrations of a compound. We determined the statistical significance using a Fisher's exact test for each of the three independent experiments and reported the largest p value of the three trials to provide a conservative estimate. In Figure 4d, Bliss Independence was assessed [30]. Neuronal survival was converted to a fractional scale with no drug treatment (17%) arbitrarily set to 0 and 100% survival set to 1. Survival for each drug and the combination was converted to a fraction and the Bliss Independence score was calculated as C = A+B−AB, where A and B are the fractional survival for LiCl and MTR and C is the predicted effect for the combination. The difference between C and the actual value (fractional) was calculated to assess synergy. LiCl had a fractional survival of 0.22, MTR 0.19 and the combination 0.51. The expected value for the combination was 0.37. The excess 0.14 was a measure of synergy.

### Quantitative Real Time PCR Analysis

qRT-PCR analyses were carried out using the SYBR Green method (BioRad) and the SmartCycler 1600 System by Cepheid Innovation. Total RNA was extracted using Trizol (Invitrogen), DNase treated (Ambion), followed by cDNA synthesis (BioRad) according to manufacturer's instructions. The primers used for amplification of the actin gene, *act-1*, are 5′-atcaccgctcttgccccatc-3′ (for) and 5′-ggccggactcgtcgtattcttg-3′ (rev). Primers for Htn transgene were previously described [3].

## Supporting Information

Figure S1 Concentration of compounds for testing in *C. elegans* was assessed using a food clearance assay. The OD of *E. coli* is reported daily for each concentration of TSA (A) and MTR (B). The mean OD is calculated for each day from triplicate samples and plotted over time.
(1.17 MB EPS)

Figure S2 *C. elegans* model of polyQ neurodegeneration and cell death [6], [10]. (A) An N-terminal fragment of human huntingtin with 150 glutamines (*Htn-Q150*) was expressed in *C. elegans* ASH neurons as previously described [6]. (B) A GFP reporter expressed in the ASH neurons was used to assess cell death; loss of GFP expression in ASH neurons indicates cell death as shown here in young *pqe-1;Htn-Q150* animals (bottom panel). (C) The ASH neuron and 5 other sensory neurons in Htn-Q150 young (top panel) or aged (bottom panel) animals were visualized by dye filling. Sensory endings of ASH neurons expressing Htn-Q150 degenerate in aged animals and fail to take up dye (bottom panel). (D) ASH neuronal survival decreases dramatically over time based on GFP fluorescence. By day 3, 75% of ASH neurons are dead in *pqe-1;Htn-Q150* animals. Error bars represent SEM. Scale bar is approximately 20 nm. (E) Only 21% of ASH neurons remain intact in aged (7 day) *pha-1* animals expressing Htn-Q150 raised at 25°C compared to young (3 day) animals based on dye filling.
(2.94 MB EPS)

Figure S3 HtnQ150 transgene levels are not decreased in the presence of LiCl or MTR. Relative HtnQ150 transgene levels were determined using the ΔΔCt method and normalized to an *act-1* control. Results are from two independent RNA collections from untreated animals (C) and animals treated with 25 mM LiCl or 0.5 mM MTR in the starved assay format for 24 hours at 15°C. The error bars indicate the standard deviation from two replicates of qRT-PCR experiments comparing untreated and drug treated samples.
(0.63 MB EPS)

Figure S4 HDAC inhibitor trichostatin A (TSA) decreases polyQ toxicity measuring ASH neurodegeneration or neuronal death. At least three trials for each efficacy assay were performed. Representative experiments are depicted. (A) TSA decreases polyQ toxicity in a *pqe-1* independent manner in *pha-1;Htn-Q150* aged animals. 30 synchronized *pha-1;Htn-Q150* L1s per well were incubated at 25°C in S medium with *E. coli* at an OD at A_595_ of 0.5 with varying concentrations of TSA to a total volume of 1 ml. On day 7, animals were collected, dye filled and 100 neurons were scored for degeneration. (B) TSA increases neuronal survival in the absence of growth using the starvation assay. 50 synchronized *pqe-1;Htn-Q150* L1s per well were incubated at 25°C in S medium with varying concentrations of TSA to a total volume of 60 µl. On day 2, the survival of 100 ASH neurons was evaluated by the presence or absence of GFP. Animals were treated with final concentration of TSA or vehicle (2% DMSO).
(0.66 MB EPS)
